# Enhancing Optogenetics‐Based Cancer Therapy Via Nanotechnology

**DOI:** 10.1002/EXP.20250399

**Published:** 2026-06-19

**Authors:** Honggang Shen, Wenzhe Yi, Rongzhang Li, Shuangshuang Hu, Xindi Qian, Dan Yan, Qian Zou, Guanru Wang, Yaping Li

**Affiliations:** ^1^ State Key Laboratory of Drug Research & Center of Pharmaceutics Shanghai Institute of Materia Medica Chinese Academy of Sciences Shanghai China; ^2^ School of Life Sciences Jilin University Changchun China; ^3^ University of Chinese Academy of Sciences Beijing China; ^4^ Shandong Laboratory of Yantai Drug Discovery Bohai Rim Advanced Research Institute for Drug Discovery Yantai China; ^5^ Yantai Kay Laboratory of Nanomedicine & Advanced Preparations Yantai Institute of Materia Medica Yantai China

**Keywords:** cancer therapy, nanotechnology, optogenetics

## Abstract

Optogenetics represents a promising frontier in precision cancer therapy by enabling spatiotemporal control over cellular behavior. However, its clinical application is limited by inefficient delivery of optogenetic components and poor tissue penetration of visible light. Recent advances in nanotechnology offer solutions to these challenges. Nanoscale drug delivery systems enhance the targeted delivery of optogenetic tools, while light‐conversion nanomaterials enable deep‐tissue activation. Besides, the integration of nanotechnology with optogenetics further facilitates the development of engineered living therapeutics, including immune cells and bacteria, allowing programmable and localized antitumor responses. Despite promising preclinical progress, key challenges remain in long‐term biosafety, immunogenicity, and precise light dosing. Future progress will depend on interdisciplinary efforts combining biocompatible nanomaterials, protein engineering, and artificial intelligence to advance clinically viable optogenetic therapies and pave the way toward personalized cancer treatment. Collectively, the synergistic integration of optogenetics and nanotechnology holds potential for overcoming longstanding barriers in cancer treatment, paving the way for precision cancer therapeutics.

## Graphical abstract

Nanotechnology enhances optogenetics‐based cancer therapy through improved delivery systems and non‐invasive optical regulation. It further enables precise and programmable control of optogenetic living therapeutics for localized antitumor responses. The integration of nanotechnology and optogenetics offers new opportunities for precise and personalized cancer treatment.

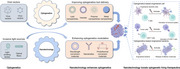

## 1 Introduction

Optogenetics, an interdisciplinary technique integrating optics and genetics, enables precise spatiotemporal manipulation of cellular activity. This approach utilizes photosensitive proteins to modulate intracellular ion dynamics and membrane potentials in response to specific wavelengths of light [1, 2, 3, 4]. Its capacity to manipulate precise spatiotemporal control of gene expression and protein activity transforms cancer treatment from a paradigm of indiscriminate cytotoxicity to one of multi‐target collaborative therapy. By precisely regulating gene expression, optogenetic systems can alleviate immune suppression, induce tumor apoptosis, modulate anticancer signal transduction, and manipulate epigenetic regulation, thereby overcoming the limitations of conventional therapies [5, 6, 7, 8, 9]. Moreover, optogenetics has facilitated the development of programmable living therapeutics, including engineered immune cells and bacteria that execute light‐controlled antitumor functions such as localized cytokine release or tumor microenvironment (TME) remodeling [10, 11, 12]. These advances provide promising tools for controllable and precise cancer treatment. However, the clinical translation of optogenetics‐based cancer therapy still faces challenges. On the one hand, there is currently a lack of effective delivery systems for optogenetic components to achieve tumor‐ specific transduction while evading immune detection [13]. On the other hand, tissue scattering and absorption restrict effective light delivery to superficial tumors, hindering applications in deep‐seated malignancies [14]. Overcoming these barriers is essential to unlock the full clinical potential of optogenetics in precision oncology.

The in‐depth development of nanotechnology provides opportunities for the development of precise and intelligent drug delivery. Nanoscale drug delivery systems (NDDSs) not only achieve biomarker‐mediated targeted drug delivery through surface engineering with tumor‐specific ligands but also protect therapeutic agents from premature degradation, thereby enhancing their pharmacokinetic profiles and therapeutic indices [15, 16]. Furthermore, the intrinsic properties of nanomaterials, such as the photothermal conversion characteristics of gold nanoparticles (AuNPs) and the immune adjuvant effects of polydopamine, provide alternative modalities for innovative cancer therapies [17, 18]. These expanding applications of nanotechnology have created opportunities to enhance optogenetics‐based cancer therapies. Firstly, engineered NDDSs (e.g., lipid‐based and polymer‐based nanoparticles) offer enhanced biosafety and expanded cargo capacity for optogenetic components [19]. Co‐delivery of optogenetic systems with auxiliary agents shows superior therapeutic effects than single components. For instance, polymer micelles can encapsulate photosensitive protein genes and light‐conversion nanomaterials. This strategy ensures the co‐localization of the optogenetic components. The light‐conversion nanomaterials convert deeply penetrating near‐infrared light into visible wavelengths, which then activate the photosensitive proteins expressed from the optogenetic cells, enabling precise optogenetic activation in deep tissues [20]. The programmable ligand conjugation also enables tumor‐specific accumulation of optogenetic systems. Besides, the inherent properties of nanomaterials provide a robust toolkit to address the challenges of spatiotemporally precise regulation in optogenetics. UCNPs transform deep‐penetrating near‐infrared (NIR, 800–1000 nm) light into visible wavelengths (450–650 nm) to activate optogenetic proteins in deep‐seated malignancies [21, 22]. Moreover, live therapeutic agents, such as engineered bacteria, can be functionalized with photosensitive nanomaterials. In this approach, bacteria are engineered as both carriers for nanoparticles (e.g., UCNPs) and as factories for producing therapeutic proteins. The nanomaterials attached to the bacterial surface act as localized transducers, converting NIR light into visible light to activate the bacteria's internal optogenetic circuits [23, 24]. This synergy enables remote, wireless spatiotemporal control over the engineered bacteria in vivo, directing them to produce and release therapeutic agents at the target site, thereby enhancing treatment specificity and controllability. This convergence creates a light‐enabled therapeutic platform for cancer therapy with deep‐tissue penetration capability and strong manipulability.

In contrast to prior reviews on optogenetics in biomedical applications, which have extensively elaborated on fundamental principles, protein engineering, and therapeutic implementations in neurology [25], metabolism [26, 27], and vision restoration [28], our review centers specifically on the synergistic integration of nanotechnology with optogenetics to enhance cancer therapy [29]. Diverging from earlier works focused on optogenetic tools in engineering and metabolic regulation, we provide a systematic analysis of nanomaterial‐based strategies designed to overcome critical translational challenges in optogenetics, such as inefficient delivery of optogenetic components and limitations in spatiotemporal control within deep tissues [30]. Furthermore, this review highlights emerging engineered living therapeutics, an area that has not been comprehensively summarized in previous publications [1, 24]. We herein survey recent advances in nanotechnology‐enhanced optogenetics for cancer treatment, categorizing and discussing innovations in NDDSs and light‐conversion nanomaterials. Based on these studies, we emphasize progress in nanotechnology‐enabled optogenetic biotherapeutic platforms, including engineered immune cells and bacteria. Finally, we outline prevailing technological challenges and future directions for the convergence of nanotechnology and optogenetics, offering insights toward the development of precise and personalized cancer therapies.

## 2 Basic Components of Optogenetics

Optogenetics synergizes optical techniques with genetic engineering to achieve precise spatiotemporal control of cellular behavior [31]. Over the past decades, optogenetics has undergone a transformative expansion, redefined as a gene encoding system that uses photosensitive protein modules to manipulate biomolecular signaling processes. This evolution has propelled optogenetics beyond neuroscience into diverse domains, including gene editing, synthetic biology, and cancer treatment [32]. A functional optogenetic system comprises three core components, including delivery vectors, optical stimulation components, and photosensitive components [33, 34, 35].

### 2.1 Delivery Vectors

**FIGURE 1:**
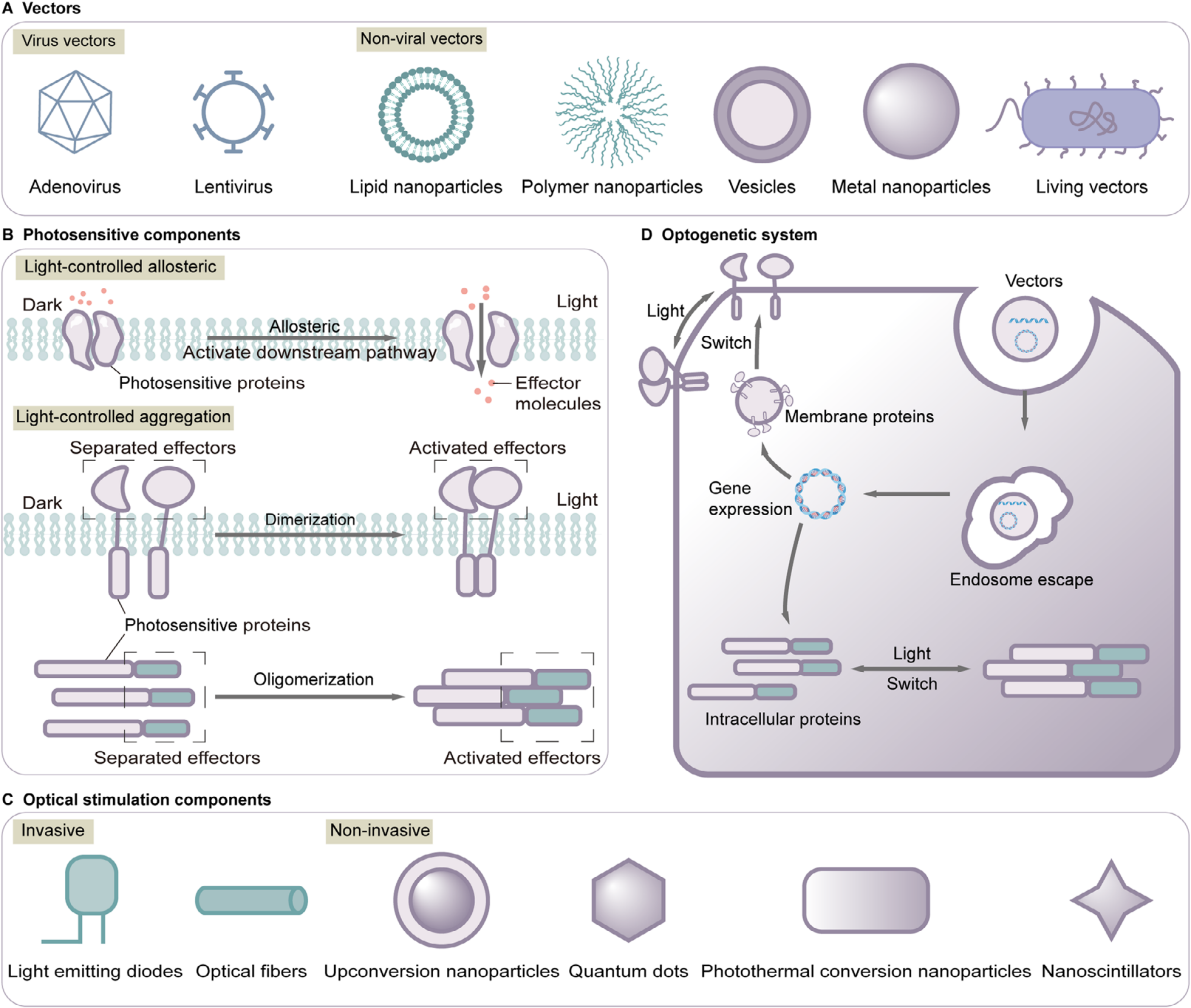
Optogenetic systems and optogenetic components. (A) Vectors. The carriers used for optogenetics are mainly divided into two categories: viral vectors and non‐viral vectors, with adeno‐associated viruses and lentiviruses accounting for the majority of viral vectors. Non‐viral vectors, including liposomes, polymers, metal particles, and living vectors. (B) Photosensitive components. Photosensitive proteins can be classified into three main categories according to their behavior after light exposure, including allosteric [40], dimerization [41, 42, 43], and oligomerization [44]. (C) Optical stimulation components. Optical stimulation components for optogenetic activation can be classified as invasive and non‐invasive. It should be mentioned that a non‐invasive system is mainly realized with the help of quantum dots, nanoscintillators, upconversion nanoparticles, etc. for distal modulation. (D) Optogenetic systems. The construction of optogenetic systems first requires the introduction of plasmids encoding the gene for photosensitive proteins into target cells, and the optical regulation of cells can be realized through photosensitive proteins when the plasmids are expressed.

In optogenetics, delivery systems serve as critical vectors for transporting optogenetic components, including photosensitive components and optical stimulation components, to target cells (Figure 1A). Viral vectors currently represent the standard in this domain, with adeno‐associated viruses (AAVs) being the most widely adopted tool [36, 37]. Although AAVs exhibit high transduction efficiency, their characteristics as viruses pose risks of unintended replication and immunogenicity. To mitigate these limitations, recombinant AAVs (rAAVs) have been engineered by deleting viral rep and cap genes, eliminating replication capacity and enhancing biosafety [38]. Complementing rAAVs, lentiviral vectors enable persistent gene expression via stable genomic integration, overcoming transient expression limitations [39].

Despite these advances, viral systems face challenges including high manufacturing costs, insertional mutagenesis risks, immune rejection potential, and restricted payload capacity [40]. Nanotechnology‐based platforms, such as lipid nanoparticles (LNPs), polymer nanoparticles, vesicles, and metal nanoparticles (MNPs), offer alternatives. These NDDSs expand deliverable payload diversity, facilitate production, and enable modular functionalization via surface engineering [15, 16, 45, 46]. Collectively, their multifunctional properties position nanomaterial‐based delivery as a transformative approach to overcoming viral vector bottlenecks in optogenetics.

### 2.2 Photosensitive Components

Photosensitive components refer to the molecular effectors, typically light‐sensitive proteins or engineered protein modules, that directly convert photonic signals into intracellular biochemical or biophysical changes. These components serve as the fundamental functional units that enable optogenetic control over cellular processes, such as ion flux, gene expression, or protein‐protein interactions, upon illumination at specific wavelengths. They are genetically encoded and expressed within target cells, constituting the core machinery that translates light input into biological output. Photosensitive components serve as the functional core of optogenetic systems, where photon absorption triggers photochemical cascades that drive conformational changes for spatiotemporal regulation. These proteins are classified into three architectural categories based on light‐responsive mechanisms (Figure 1B). Monomeric systems integrate light‐sensing and effector functions within a single polypeptide chain and achieve their biological functions through light‐induced conformational changes. For example, light–oxygen–voltage (LOV) domains, cryptochrome‐interacting basic‐helix–loop–helix (CIB1), and channelrhodopsins (ChRs) undergo structural shifts upon illumination, opening ion channels and altering cellular membrane potentials [41]. While their compact structure facilitates fusion protein design, their activation spectra are largely confined to blue light (450–500 nm), limiting deep‐tissue applications [42, 43]. Dimeric systems rely on light‐induced reversible interactions between protein pairs. These systems mostly originate from bacteria or plants, where extensive protein libraries have enabled the development of NIR‐responsive components like bacteriophytochromes (BphS), phytochrome A/phytochrome B (PhyA/PhyB), far‐red elongated hypocotyl 1 (FHY1), and phytochrome interacting factors 3/6 (PIF3/PIF6). However, the functionality of the dimeric systems requires strict spatial co‐localization of the two components, which imposes constraints on the design of the delivery vectors [44, 47, 48]. Oligomeric systems, such as cryptochrome circadian regulator 2 (CRY2) clustering module and Dronpa photo‐switch, enable light‐triggered assembly of ≥3 subunits, facilitating multi‐protein complex formation and signaling pathway regulation despite increased technical complexity [49, 50].

The optimization of photosensitive proteins is primarily based on their natural structures, with the main objective being to synthesize light‐responsive modules (Table 1). Inspired by the three natural systems, distinct classes of artificial constructs have emerged. Monomeric architectures exploit LOV domain simplicity to develop genetically encoded nanobodies undergoing reversible light‐dependent conformational changes, dynamically modulating antigen‐binding affinity to enable spatiotemporal control of intracellular signaling [51]. Dimeric systems can realize light‐induced heterodimerization, trigger Fas‐associated protein with death domain (FADD) clustering, and apoptotic pathway activation [33]. Oligomeric platforms utilize CRY2‐mediated light‐induced mixed lineage kinase domain‐like protein (MLKL) oligomerization to execute spatiotemporally precise necroptosis, demonstrating programmable cell death regulation [52]. Collectively, these optimizations expand optogenetics beyond ion regulation, enabling the execution of diverse biological functions via fusion with functional proteins.

**TABLE 1:** Examples of the photosensitive components.

Engineered proteins	Source	Activation and inactivation wavelength (nm)	Application	Refs
PhoCl fusion protein	Photocleavable protein	380/−	Light‐controlled protein regulation	[53]
OptoNBs	LOV	450/Dark	Light‐controlled protein regulation	[51]
LicV	Vivid (VVD)	450/Dark	Light‐controlled gene regulation	[54]
MLKL‐CRY2	CRY2	450/Dark	Light‐induced cell death	[46]
Fas‐CIB1‐EGFP/CRY2‐mCherry‐FADD	CRY2‐CIB1	450/Dark	Light‐induced cell death	[33]
LOVTRAP	*Avena sativa* phototropin	450/Dark	Light‐controlled protein regulation	[44]
REDMAP	PhyA‐FHY1	660/740	Light‐controlled gene regulation	[55]
PhyB protein library	PhyB‐PIF3/PIF6	660/740	Light‐controlled protein regulation	[56]
FISC	BphS	680–810/−	Light‐controlled gene regulation	[57]

### 2.3 Optical Stimulation Components

Optical stimulation components include the technological platforms and materials responsible for delivering light energy to photosensitive components with spatiotemporal precision. Unlike photosensitive proteins, optical stimulation components are external or exogenous systems that generate, transmit, or convert light. Their primary function is to provide controlled illumination to activate photosensitive proteins, thereby bridging the gap between external light sources and intracellular optogenetic machinery. As the core regulatory unit of optogenetic systems, optical stimulation components transduce light signals to precisely modulate cellular activity [58] (Figure 1C). Early‐generation systems relied on optical fibers or light‐emitting diodes (LEDs) emitting blue/yellow light. However, their limited tissue penetration depth often necessitated invasive device implantation, which carried risks of chronic inflammation and mechanical trauma [59]. Moreover, prolonged blue light irradiation causes phototoxicity through reactive oxygen species (ROS) accumulation, further restricting therapeutic applicability, particularly in deep‐seated malignancies [60, 61].

To address these limitations, next‐generation optogenetic systems have proposed some new strategies to achieve deep‐tissue optical stimulation with high spatiotemporal precision and minimal invasiveness. The first involves the use of implantable electronic devices capable of wireless light delivery in deep tissues. For instance, a recent study demonstrated an LED‐equipped electronic capsule that can be deployed in the gastrointestinal tract of pigs, enabling smartphone‐controlled light emission at depths of up to 80 mm. This system allows precise, non‐invasive optogenetic activation in deep tissues without the need for external fiber implants, thereby reducing inflammatory risks and improving translational feasibility for clinical applications [62]. For example, UCNPs act as optical transducers, converting deeply penetrating NIR light into visible blue light to activate optogenetic proteins in deep tissues [63]. Photothermal nanomaterials such as gold nanorods (AuNRs) absorb NIR light to generate localized heat, triggering thermosensitive genetic switches [18]. Additionally, bioluminescent nanoenzymes can be deployed as internally regulated light sources for therapeutic control. Upon administration of a small‐molecule luciferin substrate, these nanoenzymes catalyze a chemical reaction that produces visible light. This endogenous luminescence can then activate optogenetic components, enabling drug‐dose‐dependent regulation of cellular activities in deep tissues. This approach eliminates the need for external light delivery and provides a high degree of spatiotemporal precision for cancer therapy [64]. Collectively, these nanotechnology‐based innovations mitigate challenges inherent in conventional optogenetics, including tissue damage, insufficient penetration depth, and phototoxicity.

In summary, the classical optogenetic framework is built upon three central pillars: the delivery vector, the photosensitive components, and the optical stimulation components (Figure 1D). Consequently, the improvement of nanotechnology for optogenetics is directed at overcoming the limitations of each component to improve overall therapeutic efficacy.

## 3 NDDSs for Optogenetic Tool Delivery

### 3.1 NDDSs for Photosensitive Component Delivery

**FIGURE 2:**
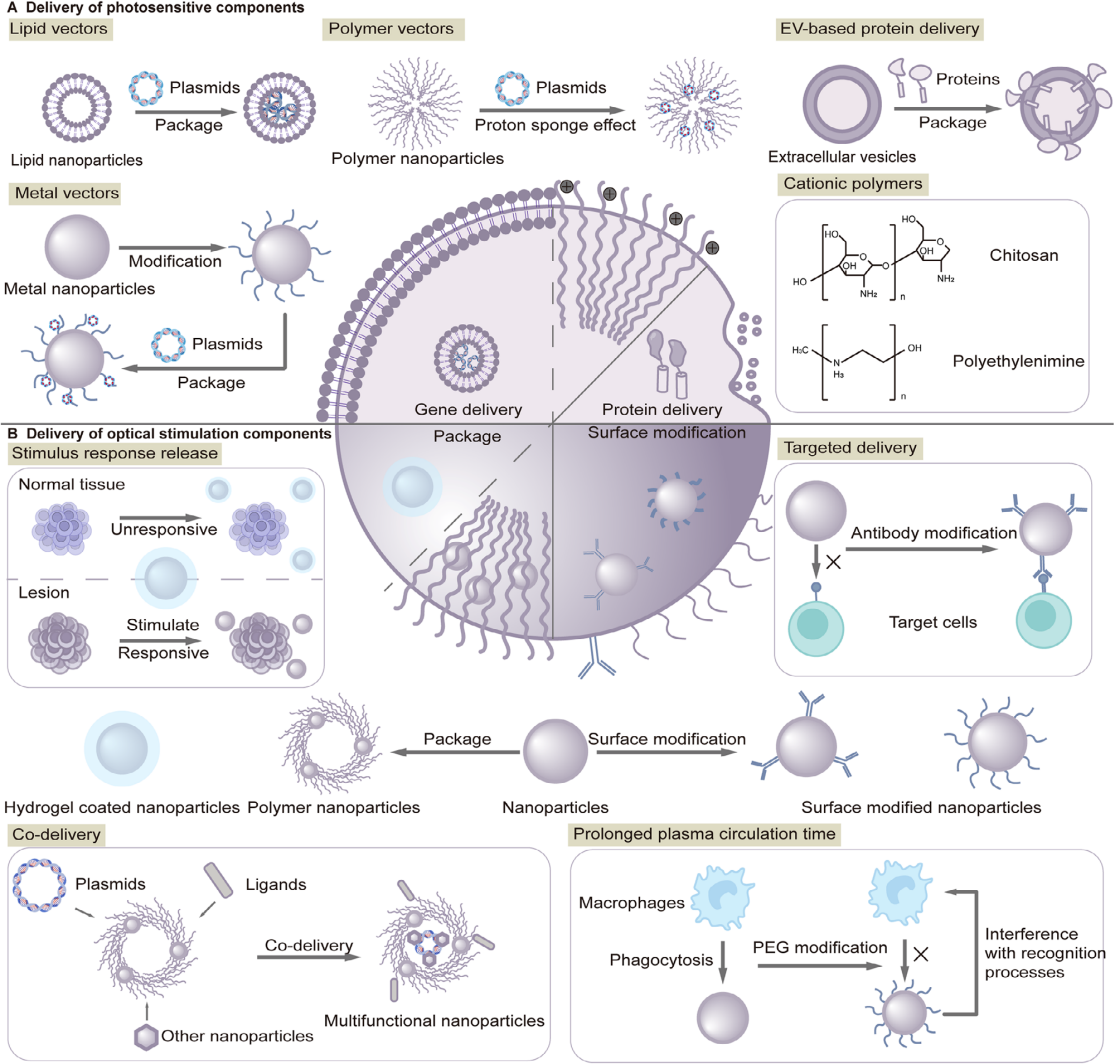
NDDSs for the delivery of optogenetic components. (A) Delivery of photosensitive components. Delivery of photosensitive protein genes can be achieved via lipid vectors, polymer vectors, and metal vectors. For lipid vectors, plasmids can be encapsulated into lipid nanoparticles, and multiple functions can be obtained by the modification of the lipid nanoparticles [19]. For polymer vectors, cationic polymers can be efficiently loaded with plasmids through the proton sponge effect and contribute to plasmid endosome escape [62, 63]. Metal vectors can obtain the ability to load nucleic acids through surface modification, which is achieved through electrostatic adsorption or covalent bonding [8]. (B) Delivery of optical stimulation components. Delivery of optical stimulation components is mainly realized by package or surface modification. Polymers can encapsulate the nanoparticles, thereby realizing stimulus–response release to reduce off‐target toxicity. It can also utilize the large loading properties to achieve co‐delivery of multiple loadings [66]. In addition, surface modification of nanoparticles can improve the targeting ability to cells and extend the plasma circulation time by modifying ligands such as PEG [67]. Nanoscale drug delivery systems (NDDSs). Polyethylene glycol (PEG).

Recent breakthroughs in nanomaterial‐based gene delivery have advanced optogenetic applications by enhancing spatiotemporal precision and biosafety (Figure 2A). Traditional viral vectors, despite their high transfection efficiency, face clinical translation barriers like immunogenicity, insertional mutagenesis risks, and limited cargo diversity [65]. In contrast, nanomaterials leverage tunable physicochemical properties and modular design to address these challenges through three carrier classes, including lipid vectors, polymer vectors, and metal vectors.

Lipid‐based vectors have emerged as promising non‐viral alternatives for optogenetic payload delivery, addressing limitations of viral platforms such as immunogenicity risks and cargo capacity constraints. Their appeal stems from their biocompatibility and engineering flexibility, which enable the modular assembly of components. Notably, preclinical studies have demonstrated high transfection efficiency using rotary evaporation‐synthesized liposomes to deliver optogenetic plasmids in vivo, achieving gene expression in lesions [19]. Beyond basic encapsulation, surface functionalization further augments targeting precision. Specifically, conjugates with hyaluronic acid (HA) or folic acid (FA) exploit receptor overexpression on diseased cells, facilitating receptor‐mediated cellular internalization with enhanced specificity. Complementing these chemical modification strategies, cell membrane‐camouflaged nanoparticles harness innate biological characteristics. By coating synthetic cores with natural membranes derived from immune cells, these nanoparticles inherit homing properties to achieve TME‐specific accumulation, where localized bioluminescent activation triggers optogenetic circuits at lesions [15]. Compared to alternative delivery platforms, LNPs offer several advantages, including well‐established and scalable manufacturing processes, such as microfluidic mixing, and high batch‐to‐batch consistency. Their soft, spherical nature and tunable size distribution improve systemic circulation [68]. However, their relatively weak structural integrity may lead to premature leakage of the encapsulated payload [69]. A positive surface charge is often engineered to promote cellular internalization, yet this must be carefully balanced against the accelerated clearance by the mononuclear phagocyte system (MPS) [70]. The metabolic pathways of lipid excipients are generally well characterized, with many components being processed via endogenous mechanisms. Nevertheless, potential excipient‐related toxicities or altered pharmacokinetic profiles due to rapid metabolism necessitate rigorous formulation screening [66, 71].

Polymer vectors leverage electrostatic interactions for efficient gene encapsulation and retention. Chitosan (CS)‐based systems achieve robust payload loading via cross‐linking with plasmid deoxyribonucleic acid (pDNA) [67]. Beyond electrostatic adsorption, cationic polymers such as polyethyleneimine (PEI) exhibit dual functionality. Their proton sponge effect facilitates nucleic acid adsorption and promotes endosomal escape [72]. This mechanism overcomes endocytic trapping of gene vectors, enhancing transfection efficiency for optogenetic component delivery. Hybrid polymer designs further integrate TME responsiveness, exemplified by redox‐sensitive PEI‐S‐S‐vitamin E succinate (VES) conjugates where disulfide bonds tether PEI to VES [46]. This system exploits elevated glutathione (GSH) levels in tumors to trigger selective disulfide cleavage, enabling controlled therapeutic release. Crucially, GSH‐mediated degradation releases encapsulated pDNA to light‐activated tumor sites, coupling optogenetic gene expression with site‐specific drug delivery. Such designs enhance transfection specificity while reducing systemic toxicity through lesion‐restricted payload release. In terms of manufacturing processes and clinical applications, polymeric nanoparticles exhibit versatility in tuning critical properties such as size, surface charge, and crystallinity, each of which significantly influences their biodistribution and targeting efficiency. Their synthesis, however, often involves complexity, requiring meticulous optimization of parameters such as molecular weight and polydispersity to ensure reproducibility [73]. A major advantage of polymeric systems lies in their tunable degradation profiles and controllable drug release kinetics, which allow for tailored therapeutic residence times, particularly advantageous for sustained applications such as optogenetics. Nevertheless, the metabolic fate of synthetic polymers and their degradation products remains less predictable compared to that of lipid‐based systems, raising concerns regarding long‐term toxicity [74]. Furthermore, the improper selection of targeting ligands can compromise the stealth functionality provided by steric stabilizers such as polyethylene glycol (PEG), potentially leading to off‐target protein corona adsorption and a consequent reduction in delivery specificity [75].

MNPs exhibit structural stability and modular capability, allowing them to acquire diverse functionalities through material modifications. Studies demonstrate that PEI‐coated UCNPs enhance plasmid transfection efficiency by facilitating electrostatic deoxyribonucleic acid (DNA) adsorption and endosomal escape mediated by the proton sponge effect [8]. Beyond electrostatic interactions, surface engineering enables stimulus‐responsive cargo release. A representative example is a UCNP‐based optogenetic system functionalized with triphenylphosphine (TPP) for mitochondrial targeting. In this design, thiol‐carboxyl coupling ensures stable nucleic acid conjugation during circulation, while ROS‐sensitive thioketal (TK) linkers allow for controlled siRNA release specifically within the mitochondria. This release is triggered by localized ROS generated from NIR‐activated photosensitizers. This strategy exhibits dose‐dependent liberation kinetics, with siRNA release rates escalating from 8% to 72% as H_2_O_2_ concentrations increase from 0 to 1.0 mM in vitro. By confining the siRNA release to the mitochondria, this strategy enhances the precision of gene silencing, thereby mitigating risks associated with siRNA leakage [76]. Collectively, this ROS‐responsive release mechanism synergizes with UCNPs' deep‐tissue penetration to enable precise optogenetic activation in vitro and in vivo. MNPs exhibit high crystallinity, structural rigidity, and controllable sizes and anisotropic morphologies (e.g., rods, plates) [18, 77]. While these attributes are beneficial for optical applications, such as imaging and phototherapy, they also introduce specific manufacturing and clinical challenges. Synthesis typically requires high‐temperature reactions and organic solvents, necessitating extensive purification steps to achieve clinical‐grade materials. The inorganic core, while metabolically stable, may persist in vivo and lead to long‐term accumulation in reticuloendothelial organs such as the liver and spleen, underscoring the need for comprehensive long‐term biodistribution and excretion studies [78, 79]. Besides, surface functionalization is essential to prevent aggregation, protect the core from degradation, and modulate protein corona formation, which critically determines the nanoparticles’ biological behavior. Therefore, appropriate surface modification will significantly impact the therapeutic efficacy of MNPs [80].

Advances in nanotechnology have enabled direct delivery of photosensitive proteins. Biologically‐derived nanostructures, particularly cellular vehicles (CVs), have emerged as promising delivery platforms. Unlike synthetic nanoparticles, CVs have gained attention as next‐generation NDDSs due to their innate biocompatibility, low immunogenicity, and ability to mimic native cell functions [81, 82]. For instance, CVs can be functionalized to display targeting ligands or engineered to encapsulate therapeutic cargo, enabling precise delivery to tumor tissues [83]. In the field of optogenetics research, studies report extracellular vehicles (EVs)‐based systems encapsulating optogenetic proteins that achieve controlled cellular activation in vitro [84]. Subsequent research achieved clustered regularly interspaced short palindromic repeat‐associated 9 nuclease (CRISPR‐Cas9) loading into EVs using light‐inducible heterodimerization systems, yielding ∼25 Cas9 molecules per EV with 51% editing efficiency in HEK293 cells and 25% in HepG2 cells [85]. For oncological applications, while protein‐delivery‐based optogenetic tumor therapy remains unreported, its demonstrated efficacy in other disease models highlights significant translational potential. The natural biological composition of CVs endows them with high biocompatibility, low immunogenicity, and the ability to target specific tissues. In contrast to synthetic nanoparticles, CVs are processed through natural metabolic pathways, providing a well‐defined excretion profile and reduced risk of accumulation [86]. However, the therapeutic use of CVs is not without risks, particularly concerning their potential role in tumorigenesis. CVs derived from certain cell types, especially those of tumor origin, may carry oncogenic proteins, nucleic acids, or miRNAs that could potentially promote tumor progression or transformation in recipient cells [87, 88]. For instance, EVs from cancer cells have been shown to transfer drug resistance traits to neighboring cells, raising concerns about the safety of using tumor‐derived CVs in therapeutic contexts [89]. Besides, scalable manufacturing remains a considerable hurdle; processes such as large‐scale production, isolation from cellular sources, and efficient cargo loading are difficult to standardize, often compromising batch‐to‐batch consistency and vesicular integrity [90]. Critical to their therapeutic application is the selection of donor cells, which dictates the surface architecture and endogenous targeting ligands, thereby requiring careful alignment with the intended disease site to maintain targeting specificity [91].

### 3.2 NDDSs for Optical Stimulation Components Delivery

Optogenetic systems require precise optical regulation for effective modulation, where nanotechnology serves as a pivotal approach to enable efficient light delivery (Table 2). Nanomaterials such as UCNPs and AuNPs leverage unique optical properties to minimize undesirable photodamage [92, 93]. However, their exogenous nature necessitates packaging or engineering to reduce immunogenicity and systemic toxicity [94].

**TABLE 2:** Examples of the application of NDDSs in the delivery of optogenetic components.

Name	Carrier type	Cargo	Indication	Refs
pG&pDTA@cRGDCL	Lipid nanoparticles	Plasmids	Melanoma	[19]
M/Lip‐FA‐NLuc@PP	Lipid nanoparticles	Plasmids	Retinoblastoma	[15]
pDTA‐Tig@CML	Lipid nanoparticles	Plasmids	Breast cancer	[67]
FAST	Polymer nanoparticles	Plasmids	Cancer	[72]
pDNA@PVHRs	Polymer nanoparticles	Plasmids	Breast cancer	[46]
UCNs@PEI	Metal nanoparticles	Plasmids	Cancer	[8]
UCNPs‐TPP/TK‐RNA	Metal nanoparticles	siRNA	Cancer	[76]
DSPs	Polymer nanoparticles	Nano‐transducers	—	[95]
CPNs	Polymer nanoparticles	Nano‐transducers	Cancer	[7]
AuNPs(PSS/PAH)2(PSS/PAH‐Dy547)	Polymer‐modified nanoparticles	Nanoparticles	—	[96]
UCNs@FA	Polymer‐modified nanoparticles	Nanoparticles	Cancer	[45]
nanoCRISPR	Polymer‐modified nanoparticles	Nanoparticles	—	[58]
Opto‐CRAC	Polymer‐modified nanoparticles	Nanoparticles	Cancer	[97]

For MNPs, surface modification is pivotal for optimizing delivery efficiency and biological interactions (Figure 2B). Ligand conjugation strategies enhance targeting specificity, exemplified by FA‐functionalized UCNPs, which demonstrate preferential tumor accumulation. In vivo imaging confirms enhanced tumor uptake of FA‐conjugated UCNPs compared to unmodified counterparts. Additionally, PEG modification extends plasma circulation time through steric stabilization effects, reducing opsonization and subsequent clearance of nanoparticles [45].

Polymer nanoparticles utilize structural versatility to enable diverse functional modifications for performance optimization. Their substantial loading capacity facilitates co‐encapsulation of multifunctional components, as demonstrated by a CS‐based platform achieving simultaneous incorporation of AuNPs and alkaline phosphatase (ALP) within a single delivery system. This approach attained 87% ALP encapsulation efficiency while maintaining structural integrity and catalytic functionality [98]. To enhance biocompatibility and circulation stability, dendronized semiconducting polymers (DSPs) incorporating PEG chains have been specifically engineered. These PEG‐modified DSPs exhibit characteristic positive zeta potential profiles and enhanced gene delivery efficiency during in vivo applications [95]. Environment‐responsive polymer materials further expand functional capabilities through spatiotemporally controlled cargo release mechanisms. pH‐sensitive hydrogels composed of sodium alginate (SA) and CS respond precisely to physiological intestinal pH gradients, triggering payload release via ionic crosslink dissociation. In vitro investigations confirmed effective cargo retention at gastric pH conditions, followed by controlled pore formation and subsequent payload release at intestinal pH, establishing precise spatiotemporal delivery of nanotechnology‐based optical stimulation components [96].

### 3.3 NDDSs for Co‐Delivery of Optogenetic Components

Recent advances have demonstrated that nanoplatforms can integrate both photosensitive components and optical stimulation modules, enabling the co‐delivery of the entire optogenetic toolkit within a single system. UCNPs represent a prototypical example: surface‐functionalized UCNPs can convert tissue‐penetrating NIR light into visible wavelengths sufficient to activate opsins, while simultaneously serving as carriers for nucleic acids or proteins through mesoporous silica coatings, polymer shells, or cleavable linkers [99, 100]. Notably, recent research has developed a UCNP‐anchored metal–organic framework (MOF) nanohybrid system that exemplifies this integrated approach. Their platform combines amine‐terminated UCNPs with pH‐responsive ZIF‐8 or ZIF‐90 MOFs, which not only deliver the VChR1 plasmid for In situ expression of cation channels but also serve as reservoirs for therapeutic metal ions (e.g., Zn^2^^+^ or Cu^2^^+^). Under NIR irradiation, the UCNPs emit green light to activate the VChR1 channels, enabling controlled ion influx and synergistic ion‐interference therapy in deep tumors [20]. Beyond nucleic acids, UCNPs have also been conjugated with functional proteins such as Cre recombinase, enabling NIR‐triggered and spatially restricted gene editing via light‐cleavable linkers [101]. Collectively, these strategies highlight the feasibility of engineering NDDSs that unify gene/protein delivery with spatiotemporally controlled light emission, providing integrated platforms for non‐invasive and precise optogenetic interventions.

## 4 Nanomaterials for Extended Optogenetic Modulation

### 4.1 Wavelength Conversion Materials

**FIGURE 3:**
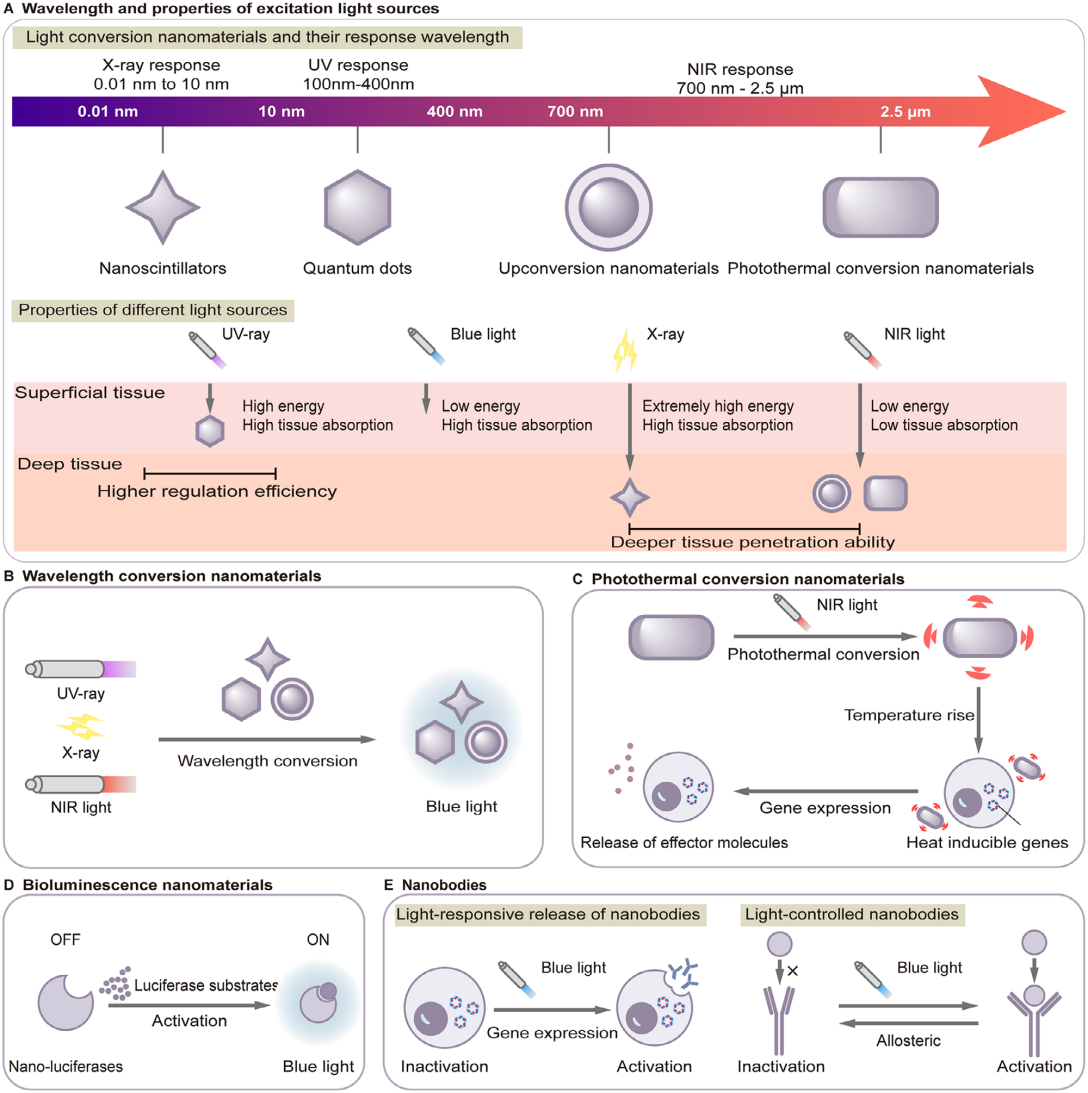
Nanotechnology for extended optogenetic modulation. (A) Wavelength and properties of excitation light sources. Different light‐converting materials have different response wavelengths, which allows them to utilize the properties of different light sources. Compared with blue light, quantum dots can utilize the high‐energy characteristics of ultraviolet (UV) light to achieve more efficient regulation of superficial tissues. X‐ray‐responsive nanoscintillators and near‐infrared (NIR)‐responsive nanoparticles can take advantage of the high penetrability to achieve regulation in the deep tissues. (B) Wavelength conversion nanomaterials can convert various lights into blue light, which can excite photosensitive components. (C) Photothermal conversion materials can absorb NIR light to generate heat, induce an increase in temperature at the lesion, which can activate the expression of thermosensitive genes, and realize therapeutic effects [100]. (D) Bioluminescent materials. Bioluminescent materials achieve luminescence through the binding of luciferase substrates to enzymes. For bioluminescent materials, the modulation of optogenetic systems is similar to chemical optogenetics [15]. (E) Nanobodies. Nanobodies can be used to regulate various biological processes through the light‐responsive release of nanobodies or light‐controlled binding of targets [103, 104].

In optogenetic applications, blue light remains the common excitation source due to its compatibility with most photosensitive proteins, despite limitations in tissue penetration depth and significant scattering within biological tissues [33, 102]. These inherent challenges have driven the development of light‐conversion nanomaterials capable of converting excitation light of specific wavelengths into emission at other wavelengths (Figure 3A). When integrated into an optogenetic system, these materials enable indirect activation using deeper penetrating or higher efficiency excitation sources while preserving spatiotemporal precision [103, 104]. Based on excitation characteristics, these systems are categorized as X‐ray‐responsive, ultraviolet (UV)‐responsive, and NIR‐responsive (Figure 3B).

X‐ray‐responsive systems (0.01–10 nm) enable synergistic integration of high‐energy radiation with optogenetic control, utilizing nanoscintillators to achieve exceptional spatial resolution for deep‐tissue activation [105]. These materials serve as optical transducers, converting X‐ray energy into visible‐spectrum emissions. Studies utilizing cerium‐doped gadolinium aluminum gallium garnet (Ce:GAGG) nanoscintillators demonstrated yellow light emission (peak ∼520–530 nm) with a yield of 46,000 photons MeV^−1^ upon X‐ray irradiation, achieving successful neuronal modulation in murine models at a dose rate of 1.0 Gy min^−1^, which produced a local light intensity of approximately 2 µW cm^−2^ near the implant site [106]. Research employing europium‐doped gadolinium tungstate (Gd_2_(WO_4_)_3_:Eu) nanoparticles documented red luminescence (peak ∼613 nm) under X‐ray excitation, enabling transcranial optogenetic stimulation of cortical neurons expressing red‐shifted channelrhodopsin [107]. For the blue spectrum essential to most photosensitive proteins, investigations with lutetium oxyorthosilicate doped with cerium (LSO:Ce) confirmed blue light emission capable of activating ChR2 in hippocampal slices, effectively modulating synaptic transmission dynamics. The LSO:Ce scintillator exhibits a yield of approximately 30,000 photons MeV^−1^. In the referenced study, stimulation of LSO:Ce particles with a power density of 300 µW cm^−2^ elicited measurable photocurrents (−22.4 ± −2.3 pA) in ChR2‐expressing neurons, demonstrating its efficacy as a light source for optogenetics [108].

UV‐responsive systems (100–400 nm) primarily utilize quantum dots (QDs), 1–10 nm semiconductor nanocrystals characterized by broad excitation spectra spanning UV–visible wavelengths and narrow emission bandwidths (20–50 nm) [109]. These distinctive photophysical properties establish QDs as ideal candidates for high‐resolution cellular imaging and multiplexed labeling applications within optogenetic systems [110]. Historically, Group II–VI QDs (e.g., CdSe, CdTe, ZnS) have enabled light‐gated activation of neuronal ion channels in foundational optogenetic studies [111]. However, their biomedical application, particularly in long‐term or implantable settings, is significantly constrained by biocompatibility concerns. The potential leaching of cytotoxic heavy metal ions (e.g., Cd^2^^+^) from these QDs poses substantial risks of oxidative stress, inflammatory responses, and organ accumulation, primarily in the liver and spleen, which may lead to chronic toxicity and impede clinical translation [112, 113]. Consequently, these concerns have accelerated the development of biocompatible Group IV alternatives, including silicon QDs and carbon QDs, which demonstrate enhanced bio‐inertness and lower cytotoxicity [114, 115]. Emerging graphene QDs exhibit particular promise through their exceptional biocompatibility and photostability, effectively addressing both toxicity limitations and spectral requirements [116]. Notably, UV‐responsive QDs are extensively applied in photodynamic therapy (PDT). As exemplified by platforms such as the IR820‐conjugated system, which demonstrates a photothermal conversion efficiency of 46.0% under 808 nm laser irradiation at 1 W cm^−2^. Their convergence with nanotechnology‐based optogenetics holds potential promise for developing integrated therapeutic platforms where targeted light activation and photodynamic action synergize [117].

NIR‐responsive systems (700 nm–2.5 µm) leverage the unique photonic advantages of this spectral region, including exceptional tissue penetration depth and low phototoxicity, rendering them particularly promising for deep‐tissue optogenetic applications [118]. However, the practical implementation of such systems faces a critical challenge: most optogenetic components require visible light activation (400–650 nm), which exhibits limited tissue penetrance. This spectral mismatch necessitates efficient wavelength conversion strategies. Herein, UCNPs offer a solution. Specifically, the luminescent properties of UCNPs are governed by doped rare earth ions, wherein sensitizers (e.g., Yb^3^^+^) absorb low‐energy NIR photons and subsequently transfer energy to activator ions (e.g., Er^3^^+^, Tm^3^^+^, Ho^3^^+^) through non‐radiative processes. Critically, specific dopant combinations enable precise wavelength conversion, effectively bridging the NIR‐to‐visible gap. For instance, Yb^3^^+^–Er^3^^+^ co‐doping facilitates efficient 980 nm to 550 nm upconversion, whereas Yb^3^^+^–Tm^3^^+^ configurations achieve 800 nm to 475 nm transitions. These conversions collectively cover the activation spectra required for most optogenetic components, thereby enabling NIR‐driven photomodulation in deep tissues [119]. However, the clinical translation of UCNPs is contingent upon addressing concerns regarding their long‐term biosafety and biocompatibility. The incorporation of heavy lanthanide ions raises potential issues related to ion leakage, chronic accumulation in reticuloendothelial organs, and delayed clearance, which may provoke oxidative stress, inflammatory responses, or unforeseen immunogenicity over extended periods [120]. To mitigate these risks, substantial efforts have been devoted to surface engineering strategies, which not only enhance targeting specificity but also reduce ion leakage and improve biocompatibility [121].

The performance of UCNPs is optimized through surface engineering approaches. Targeting capabilities are enhanced via conjugation strategies, as demonstrated by streptavidin‐functionalized UCNPs (UCNPs‐Stv) that enable precise dendritic cell (DC) adjustment when integrated with optogenetic calcium release‐activated calcium (Opto‐CRAC) platforms. For instance, UCNPs‐Stv enabled NIR‐triggered Ca^2^^+^ influx in DCs upon 980 nm laser irradiation at a power density of 30 Mw mm^−2^ [97]. Loading capacity and transfection efficiency improvements employ polymer modifications, wherein PEI‐coated UCNPs (UCNs@PEI) with an average diameter of ∼35 nm exhibit enhanced pDNA adsorption through electrostatic interactions while promoting endosomal escape via the proton sponge effect. Furthermore, these UCNs@PEI nanoparticles, when excited by a 980 nm NIR laser at a power density of 4 W, emit blue light at ∼475 nm, effectively activating optogenetic proteins [8]. Furthermore, polymer coatings simultaneously address biocompatibility requirements. Alternative biocompatibility strategies include CS/SA layer‐by‐layer assemblies constructing hybrid microsphere platforms for efficient encapsulation of upconversion microcolloids [122]. Although lanthanide‐doped systems have stable wavelength conversion capabilities, their inherent toxicity still inevitably limits their clinical use. Beyond conventional lanthanide‐doped systems, emerging triplet‐triplet annihilation upconversion platforms represent innovations. These molecularly engineered complexes achieve stable NIR‐to‐blue photoconversion through controlled triplet energy transfer mechanisms, thereby establishing novel paradigms for deep‐tissue optogenetic activation [123].

The selection of optical stimulation components is dictated by specific application requirements. X‐ray systems provide exceptional spatial precision but have potential radiation toxicity. UV systems enable efficient modulation of superficial tissues, but the depth of tissue penetration is limited. NIR systems excel in tissue penetration depth and exhibit favorable safety profiles, but the quantum yield of nanoparticles used for NIR conversion needs to be improved. Ultimately, optimal selection depends on three key parameters: target tissue depth, required spatial resolution, and acceptable security thresholds (Table 3).

**TABLE 3:** Comparison of different systems for optogenetic modulation.

System	Wavelength range	Advantages	Limitations	Applications	Refs
X‐ray‐responsive	0.01–10 nm	• Exceptional spatial resolution • Deep tissue penetration	• Potential radiotoxicity and DNA damage • Requires heavy shielding and safety protocols • Limited availability of X‐ray sources for routine use	• Regulation in deep tissues • Bioimaging • Radiotherapy	[105, 106, 107]
UV‐responsive	100–400 nm	• Enables efficient activation • Suitable QDs with narrow emission	• Shallow tissue penetration • High phototoxicity and ROS generation • Risk of cadmium leaching QDs	• Superficial tumors • Photothermal therapy • Bioimaging	[124, 125, 126, 127]
NIR‐responsive	700 nm–2.5 µm	• Deep tissue penetration • Low phototoxicity and scattering • Non‐invasive and biocompatible excitation	• Low quantum yield of UCNPs requires high power or dose • Potential long‐term accumulation of lanthanides • Limited activation spectrum for certain opsins	• Deep‐tissue optogenetics • Cell modulation • Combined living therapy	[21, 22, 60, 76]

### 4.2 Photothermal Conversion Materials

Photothermal conversion nanomaterials enable efficient transformation of photon energy into localized thermal energy, a capability garnering research interest within nanotechnology‐based optogenetics (Figure 3C). Three distinct nanoparticle classes have emerged as promising platforms for optogenetic applications, each exploiting distinct photothermal conversion mechanisms.

Metal nanomaterials, particularly gold‐based nanostructures, have become cornerstone elements in optical modulation technologies due to their photonic properties, most notably tunable localized surface plasmon resonance (LSPR) effects. Unlike organic photosensitizers, these inorganic systems offer photostability and engineerable resonance peaks. Under NIR irradiation, AuNPs exhibit strong photon absorption and efficient conversion into thermal energy, resulting in localized hyperthermia within specific tissues [128, 129]. This photothermal effect has been utilized for the controlled release of genetic and pharmaceutical agents. For instance, AuNRs have been employed as photothermal transducers to trigger the release of DNA‐based agonists, whereby LSPR‐mediated heating disrupts thermostable bonds in carrier matrices. One study demonstrated that approximately 50% of therapeutic DNA was released within 4 min of NIR exposure at a physiologically tolerable temperature of 44°C, allowing for spatiotemporally controlled gene delivery with minimal off‐target effects [18]. This precise spatiotemporal control establishes metal nanomaterials as efficient tools for triggered delivery systems.

Conjugated polymer nanoparticles (CPNs) represent promising photothermal platforms that integrate high conversion efficiency with biological compatibility. Their π‐conjugated backbones enable strong NIR absorption and promote non‐radiative decay [130, 131], as exemplified by aza‐heterocycle‐based donor–acceptor polymers such as PBABDF‐TVT, which exhibit mass extinction coefficients up to ∼55 cm^−^^1^·mg^−^^1^·mL and photothermal conversion efficiencies of ∼40.7% under 808 nm irradiation [132]. Indeed, tightly packed and well‐ordered π‐stacked structures within CPNs facilitate exciton delocalization and open efficient non‐radiative relaxation channels, thereby suppressing radiative emission and enhancing heat generation. This structure–function correlation has been consistently observed across numerous studies, linking greater backbone planarity, closer π–π contacts, and higher crystallinity to fluorescence quenching and improved photothermal conversion efficiency [133, 134]. In contrast, twisted or disordered polymer conformations and amorphous aggregation states restrict exciton delocalization, promote intersystem crossing, and facilitate energy transfer to molecular oxygen, thereby enhancing the generation of singlet oxygen and other ROS [135, 136]. Capitalizing on these structure–property relationships, recent studies have incorporated CPNs as photothermal transducers in NIR‐activated therapeutic platforms. For instance, Li et al. demonstrated that quinoid CPNs (PIS NPs) with a strong NIR‐II absorption peak at 1026 nm could efficiently convert 1064 nm laser energy into heat, achieving a temperature rise of over 40°C in solution and nearly 20°C in tumors. This localized hyperthermia directly induced tumor cell necrosis and apoptosis, leading to significant tumor growth inhibition [137]. Beyond direct thermal cytotoxicity, CPN‐mediated hyperthermia also initiates downstream immunological effects. Under 808 nm irradiation, CPNs can raise local temperatures to approximately 57°C, inducing heat‐shock protein 70 (HSP70)‐dependent interferon‐*γ* (IFN‐*γ*) secretion and reprogramming tumor‐associated macrophages toward an M1 phenotype [7]. This approach demonstrated synergistic ablation‐immunomodulation effects, establishing CPNs as versatile platforms for remotely controlled combinatorial therapy.

Semiconductor nanoparticles (SPNs) exhibit high photothermal conversion efficiencies, with both absorption spectra and thermal effects precisely tunable through rational polymer backbone design and side‐chain modifications [138]. One strategy involves incorporating gene regulation into the photothermal process. For example, an SPN‐based system (SPNHT) was developed to encapsulate an HSP70 promoter‐driven plasmid encoding a TNF‐related apoptosis‐inducing ligand (TRAIL) transgene. Upon 1064 nm laser irradiation, SPNHT achieved local temperatures of approximately 70°C, resulting in direct cytotoxic effects and concurrent activation of TRAIL expression via the HSP70 promoter. This process promoted apoptosis even in thermoresistant tumor cells. Compared to conventional near‐infrared I (NIR‐I) agents, this NIR‐II‐optimized system improved tissue penetration while maintaining high photothermal conversion efficiency [139]. These findings demonstrate that SPNs can synergize localized hyperthermia with stress‐induced gene expression to produce combined thermal and genetic antitumor responses.

### 4.3 Bioluminescent Materials

Bioluminescent systems offer a practical alternative to external light sources by utilizing endogenous bioluminescence to drive optogenetic activation [64] (Figure 3D). This approach addresses inherent limitations of external illumination, particularly regarding tissue penetration depth and phototoxicity. In a representative platform, nanoluciferase (NanoLuc) is integrated with photosensitive CRY2‐CIB1 complexes. Operationally, injection of the NanoLuc substrate initiates blue light emission, with emission intensity directly adjustable through substrate concentration modulation. This tunability enables dose‐dependent control of optogenetic activation. Critically, in vivo studies confirm that this system significantly reduces retinal photodamage compared to external blue‐light irradiation while maintaining equivalent CRY2‐CIB1 heterodimerization activation efficiency [15]. The preservation of activation efficacy alongside enhanced biocompatibility validates the dual‐functional advantage of bioluminescent platforms for in vivo optogenetic applications.

### 4.4 Nanobodies

An increasing array of optogenetic tools have been developed to reversibly control interactions between engineered protein domains. However, tools enabling light‐switchable binding to untagged target proteins of interest remain limited. Nanobodies, as versatile protein binders with target specificity, exhibit advantages when integrated with optogenetics [140, 141]. This integration presents dual advantages, including enhancing the spatiotemporal specificity regulation of nanobodies and designing efficient light‐controlled nanobodies (Figure 3E).

In combination therapy, research has established a platform integrating light‐activated split Cas9 gene editing with optogenetic protein degradation. Under blue light, this system executes nuclear genome editing of the *Survivin* gene and nanobody‐mediated degradation of *Survivin* proteins. In vivo evaluation using UMUC‐3 tumor models demonstrated 35% tumor cell apoptosis, surpassing monotherapeutic approaches [142]. Complementary studies demonstrate light‐triggered nanobody secretion systems, where engineered microbial chassis secrete programmed death ligand 1 (PD‐L1) nanobodies in response to green light illumination. Quantification confirmed secretion of 6630 pg mL^−1^ PD‐L1 nanobodies under stimulation, achieving 16% tumor cell binding efficiency. In 4T1 breast cancer models, this approach synergistically enhanced photodynamic therapy, significantly promoting cytotoxic T cell activation [143].

The design of light‐responsive nanobodies centers on the fusion of photosensitive domains with functional binding modules. Research has engineered optogenetic nanobodies (OptoNBs) through LOV domain integration, enabling reversible photo‐switchable binding to endogenous targets for pathway modulation. In HEK293 cells, OptoNBs exhibited illumination‐dependent cytosolic relocalization, with the LaM8‐AK74 variant demonstrating a 40% fluorescence intensity shift between dark and illuminated states, performance comparable to established optogenetic tools [51, 144]. Building on this foundation, an optimized optogenetic monomer (OptoMB) was developed, featuring a 330‐fold illumination‐dependent affinity shift. Bio‐layer interferometry quantified this transition; the dark‐state dissociation constant is 0.19 ± 0.11 µM versus the illuminated‐state is 63 ± 23 µM, confirming reversible target engagement through allosteric conformational changes [145]. Furthermore, modular engineering allows these systems to adapt to diverse targets. Recent work expanded Trim‐Away technology through CRY2 domain fusion, creating optogenetic Trim‐Away (optoTrim‐Away) for light‐controlled protein degradation. Live‐cell imaging confirmed rapid degradation of tagged proteins within 1 h, achieving over 70% degradation efficiency and validating its precision control capabilities [146].

## 5 Nanotechnology Boosts Optogenetic Living Therapeutics

### 5.1 Optogenetics‐Based Engineered Cell

Immune cells serve as central effectors in cancer immunotherapies through their inherent capacity to recognize and eliminate malignant cells, thereby suppressing tumor initiation and progression. However, the immunosuppressive TME frequently compromises their tumoricidal activity [147]. Nanotechnology‐based optogenetic engineering presents the methods to overcome this limitation by enabling spatiotemporally precise modulation of immune functions via light‐responsive genetic circuits. This approach not only enhances therapeutic efficiency but also minimizes systemic toxicities through localized activation.

**FIGURE 4:**
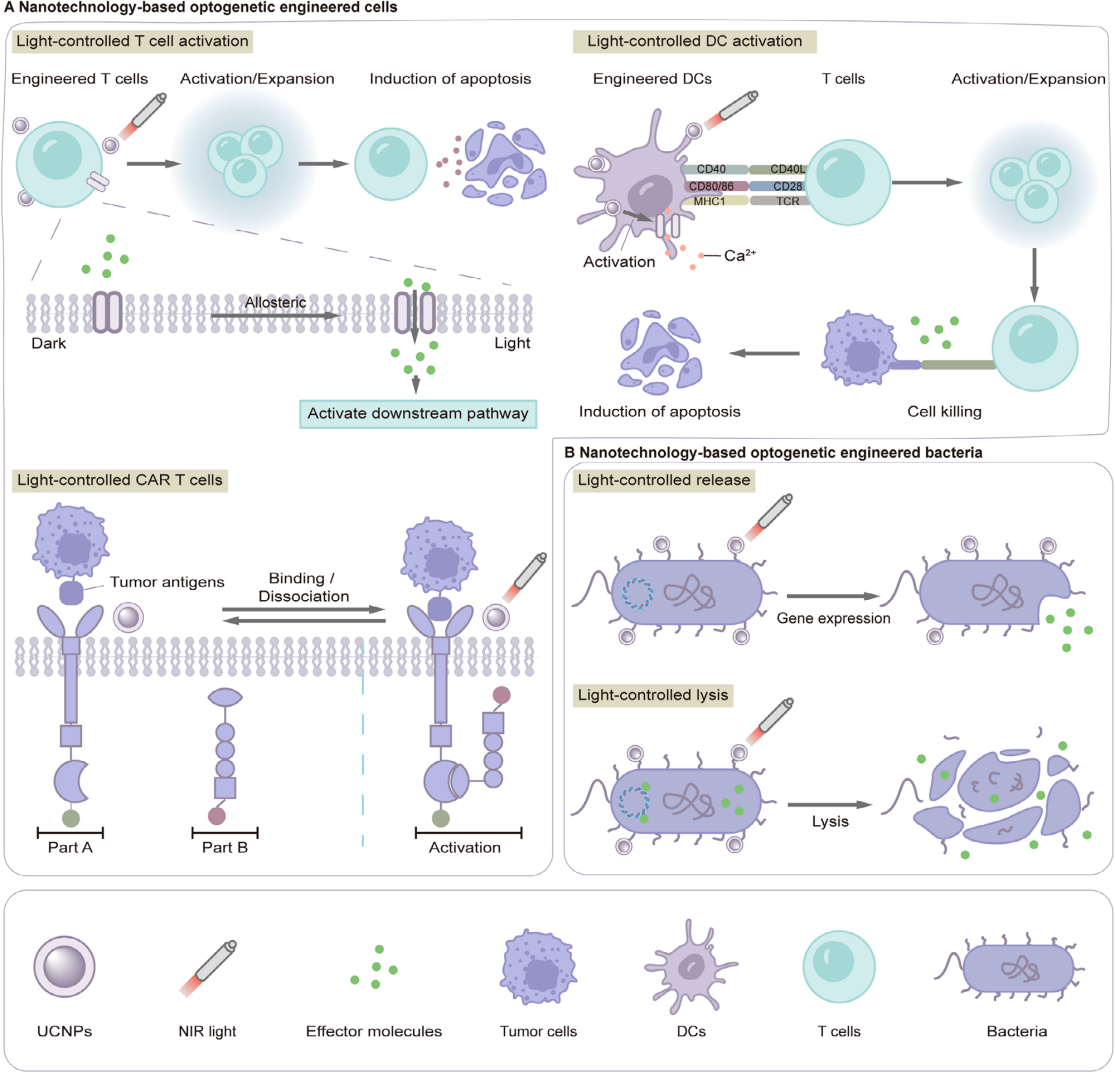
Nanotechnology‐based optogenetic living therapeutics. (A) Nanotechnology‐based optogenetically engineered cells. The introduction of photosensitive proteins into engineered cells can be used to activate immune cells using light‐controlled ion channels for immunomodulation [110, 112]. By splitting CAR elements and fusing them with photosensitive protein pairs, light‐controlled CAR T cells can be constructed to realize the control of CAR T cells [118]. (B) Nanotechnology‐based optogenetically engineered bacteria. Light‐controlled bacteria can secrete proteins under light conditions [122]. In addition, the light‐induced lysis of the bacteria allows the release of cargos from the bacteria, which can induce the immune response [23]. DCs, dendritic cells; UCNPs, upconversion nanoparticles; CAR, chimeric antigen receptor; NIR, near‐infrared.

The antitumor principles of optogenetics‐based engineered immune cells depend on their inherent functional properties, which exhibit cell‐type specificity (Figure 4A). T cell immunotherapies demonstrate limited effectiveness against solid malignancies due to microenvironmental suppression of infiltrating lymphocytes. Optogenetic control relieves this limitation by enabling non‐invasive, real‐time modulation of T cell activity. Research has developed light‐responsive circuits where blue‐light stimulation induces engineered T cells to secrete interleukin‐2 (IL‐2), interleukin‐15 (IL‐15), and tumor necrosis factor‐*α* (TNF‐α), prolonging survival in tumor‐bearing mouse models [148]. To overcome blue light's penetration constraints, alternative approaches employ PhyB‐based engineered T cell systems, achieving reversible NIR‐light activation with calcium influx peaking at 250 s post‐stimulation [149]. However, current strategies for engineering T cells often rely on viral vectors for the stable genomic integration of optogenetic components, such as light‐gated ion channels or dimerization systems. These methods face challenges, including insertional mutagenesis, immunogenicity, and limited cargo capacity. Non‐viral alternatives, such as LNPs or polymer‐based carriers, offer improved biosafety and targeting through surface functionalization. Nevertheless, achieving efficient and specific transduction in vivo remains a hurdle [100]. Additionally, premature activation of engineered T cells during manufacturing or circulation could lead to exhaustion or off‐target effects, underscoring the need for tightly controlled expression and delivery systems [11].

Beyond T cells, DCs have been engineered to amplify antitumor immunity through optogenetically enhanced antigen presentation [150]. Advanced platforms integrate nanotechnology with optogenetics, where NIR‐irradiated UCNPs emit localized blue light to activate photosensitive calcium channels in DCs. This triggers Ca^2^^+^ influx, potentiating nuclear factor of activated T cells translocation and enhancing cross‐presentation of tumor antigens. In melanoma models, NIR stimulation significantly suppressed primary tumor growth and reduced pulmonary metastasis, demonstrating 40% fewer tumor foci in lungs alongside diminished tumor volumes [97, 151]. Despite these advances, targeting DCs presents specific challenges. DC‐specific optogenetic modulation often involves the use of NDDSs decorated with DC‐targeting ligands to deliver optogenetic components. However, risks include off‐target activation of other antigen‐presenting cells [152]. Moreover, the heterogeneity of DC subsets (e.g., cDC1 vs. cDC2) necessitates specific targeting strategies to avoid unintended immunosuppressive outcomes [153]. Besides, variability in DC maturation status and TME‐driven dysfunction may also limit the efficacy of optogenetics‐based engineered cells, requiring combination approaches to ensure robust immune activation [154].

Optogenetic engineering of chimeric antigen receptor (CAR) T cells represents a new strategy for spatiotemporally precise immunotherapy. This approach mitigates life‐threatening complications of conventional CAR T cell therapies through light‐controlled modulation of T cell activity, notably cytokine release syndrome (CRS) characterized by interleukin‐6 (IL‐6) and IFN‐*γ* dysregulation [155, 156]. Blue‐light‐responsive systems exemplified by optogenetic CAR (optoCAR) utilize light‐inducible heterodimerization modules to enable transient assembly of functional CAR complexes [157, 158, 159]. These designs confer light‐responsive activation, such as the illumination‐induced CD69 upregulation, extracellular signal‐regulated kinases phosphorylation, and downstream nuclear factor kappa‐B (NF‐κB) activation in primary human T cells [160]. Innovative combinatorial platforms enhance functional versatility. The light‐inducible nuclear translocation and dimerization (LINTAD) system integrates CRY2‐CIB1 with a light‐inducible nuclear localization signal (biLINuS) to enable spatiotemporal regulation of transcriptional programs. Blue light stimulation of LINTAD‐engineered CAR T cells induces cytotoxicity against target tumor cells in vitro, while in vivo studies demonstrate tumor growth suppression through coordinated nuclear translocation of therapeutic genes and enhanced T cell functions [48].

For deep‐tissue therapeutic applications, research has engineered NIR‐responsive CAR systems utilizing NIR‐responsive photodimerization modules that self‐assemble into functional CARs under 808 nm illumination. This approach achieved antitumor activity in preclinical models with precise spatiotemporal control [161]. Concurrent nanotechnology strategies significantly enhance light delivery efficiency, exemplified by UCNPs conjugation to CAR T cell membranes, enabling NIR‐triggered activation in deep‐seated malignancies. The UCNPs convert incident NIR light into localized blue emissions, activating optoCAR modules while maintaining spatial precision [11]. Complementary approaches employ photothermal nanomaterials as transducers. AuNRs serve as efficient transducers that convert NIR energy into hyperthermia, actuating temperature‐sensitive genetic switches. This thermal induction mechanism drives controlled expression of CAR components via heat‐shock promoters, significantly enhancing tumor‐specific killing [162]. While these optogenetic strategies effectively address the penetration limitation of traditional optogenetics, they introduce distinct sets of challenges. The conjugation of UCNPs to T cell membranes must be efficient and stable without compromising cell viability, migration, or effector functions [163]. Furthermore, potential nanoparticle detachment and uncertain long‐term fate in vivo raise concerns about systemic exposure to inorganic materials, which could lead to unforeseen immunogenicity or off‐target accumulation [164]. Similarly, photothermal approaches require precise thermal control, as inhomogeneous heat distribution may result in inadvertently triggering pro‐inflammatory effects or heat shock responses in adjacent healthy tissues, potentially causing off‐tumor toxicity [165]. Furthermore, to meet clinical application requirements, engineered cells themselves also require the implementation of stringent biosafety measures to mitigate risks such as uncontrolled proliferation and immunogenicity. The key to avoiding these adverse effects lies in the circuit design of the cells. For instance, incorporating safety switches, such as inducible apoptosis genes, enables rapid elimination of engineered cells in case of adverse events like CRS [52, 166].

### 5.2 Optogenetics‐Based Engineered Bacteria

In synthetic biology, engineered bacteria are often conceptualized as “drug factories” due to their capacity for therapeutic drug production. The core functionality of these systems hinges on precisely designed biological circuits that control gene expression dynamics, which coincides with optogenetic regulation. Indeed, optogenetically engineered bacteria, constructed via synthetic biology methodologies, exhibit light‐specific responsiveness to modulate gene expression or cellular functions with high precision (Figure 4B). Beyond basic genetic control, these microbial platforms demonstrate enhanced programmability, which permits the integration of multifunctional modules while preserving adaptability to dynamic TME conditions. Building upon this programmability, nanotechnology‐integrated optogenetic living therapeutics achieve spatiotemporal control. Specifically, through light‐triggered mechanisms such as controlled cargo secretion and programmed cellular lysis, they enable on‐demand release of therapeutic payloads at targeted sites and timescales, thereby maximizing treatment specificity while minimizing systemic toxicity.

For controlled therapeutic release, optogenetics‐based engineered bacteria function as targeted gene delivery vectors specifically adapted to hypoxic TMEs. These systems typically incorporate light‐activated secretion circuits, with studies demonstrating *Escherichia coli* (*E. coli*) engineered to secrete deoxyviolacein under blue‐light induction, inhibiting tumor growth in vitro while maintaining sustained therapeutic production for 42 days [167]. To overcome deep‐tissue penetration limitations, research has developed a UCNPs‐conjugated bacteria system that converts NIR to blue light, enabling remote activation of IFN‐*γ* secretion. In B16F10 melanoma models, this platform reduced tumor volume through IFN‐*γ*‐mediated systemic immune activation, with serum cytokine levels exceeding controls [168]. Advanced photothermal hybrid systems combine optogenetics with photodynamic therapy, enabling concurrent *α*‐hemolysin release and tumor‐destructive hyperthermia under NIR irradiation [12].

Controlled lysis systems represent a precision therapeutic strategy by employing programmed cellular disintegration for targeted payload release. Building upon engineered bacterial chassis described previously, these systems incorporate optogenetic circuits activated by specific NIR wavelengths to induce two critical events: bacterial lysis and TRAIL expression. Upon optical stimulation, rapid cellular disruption ensures immediate liberation of pre‐synthesized TRAIL proteins, which directly initiate caspase‐mediated apoptosis in tumor cells through death receptor binding [23]. Importantly, this lysis mechanism simultaneously enhances biosafety via integrated post‐therapeutic clearance protocols. Specifically, subsequent light exposure triggers complete bacterial elimination after payload delivery, thereby preventing off‐target colonization and potential inflammatory complications [16]. Collectively, these designs address dual imperatives within a single integrated platform, establishing a robust foundation for clinical translation of living therapeutics.

Emerging frontiers in optogenetic engineering now target the gut microbiota as a programmable therapeutic interface. This approach capitalizes on the gut's unique accessibility to light‐based interventions and its critical role in systemic health. Central to this paradigm is the gut‐brain axis, a bidirectional communication network wherein microbial metabolites directly regulate neurological functions. Inspired by this nexus, light‐controllable bacterial systems have been engineered to treat neuropsychiatric disorders through precise modulation of neuroactive molecules [169, 170]. Notably, research demonstrates that UCNP‐integrated platforms can effectively ameliorate anxiety‐like behaviors and mitigate Parkinson's disease pathology by remotely regulating specific microbial metabolic pathways [171]. Beyond neurological applications, given the gut microbiome's established characterization as a “second genome” with profound oncogenic associations [126], optogenetic gut microbial interventions represent an emerging frontier. Such strategies offer potential for both cancer prevention via microbiome normalization and precision oncology through targeted anti‐tumor effector delivery, thereby bridging microbial with clinical therapeutics.

Although some bacteria can naturally target hypoxic tumor tissues, the therapeutic application of engineered bacteria introduces biosafety concerns [172]. Key risks include horizontal gene transfer of engineered plasmids to commensal or pathogenic flora, potential immunogenic reactions such as sepsis or cytokine storms due to bacterial component recognition, and unintended long‐term colonization leading to chronic inflammation or metabolic disruption [173]. To address these challenges, the design of biosafety circuits is essential. For instance, auxotrophy‐based systems ensure bacterial dependence on exogenous nutrients unavailable in human tissues, preventing uncontrolled proliferation [174]. Additionally, engineered kill switches can be activated by external triggers to eliminate bacteria after therapy [175]. Besides, nanotechnology can further enhance these strategies by facilitating the targeted delivery of optogenetic circuits and safety modules, ensuring robust control of engineered bacteria [176].

## 6 Conclusion and Outlook

Optogenetics enables the transition of cancer treatment from conventional cytotoxic interventions to programmable precision medicine. The integration of nanotechnology with optogenetics creates innovative therapeutic platforms that merge genetic specificity with photonic controllability. Non‐viral delivery vectors, including lipid‐based nanoparticles, polymers, and metallic nanostructures, enable efficient co‐delivery of optogenetic components [67]. Surface modification further achieves tumor‐specific targeting through receptor–ligand interactions. Engineered nanomaterials also serve as multifunctional photonic mediators that enhance optogenetic activation through extending light penetration depth and improving spatial precision. These nanoparticles penetrate deep into the tumor tissue and convert deeply penetrant NIR to localized visible light (480–650 nm), overcoming traditional limitations in tissue accessibility. Compared to implanted light sources, they minimize inflammatory responses and mobility disturbances [177]. In adoptive cell therapies, optogenetic systems allow precise parameterization of light exposure to activate engineered immune cells or bacteria. Such dynamic control initiates predetermined biological responses, thereby optimizing therapeutic accuracy while reducing systemic toxicity [148]. Furthermore, the integration of nanotechnology with optogenetics offers promising strategies to overcome specific barriers imposed by the TME, such as hypoxia, acidic pH, elevated interstitial fluid pressure, and immunosuppressive cues. NDDSs can be engineered to respond to TME‐specific stimuli (e.g., pH‐sensitive release, enzyme‐triggered activation) to enhance localized drug delivery and optogenetic activation. For instance, redox‐sensitive polymers facilitate GSH‐mediated payload release in the TME, while HA‐coated nanoparticles promote CD44 receptor‐mediated uptake in tumor cells. Additionally, nanotechnology‐enabled modulation of the TME can synergize with optogenetic therapies to improve treatment outcomes.

Although the convergence of nanotechnology and optogenetics for cancer therapeutics is promising, it still faces considerable challenges, particularly when compared to conventional viral vector‐based systems. While viral vectors offer high transduction efficiency and sustained transgene expression, their clinical translation is limited by immunogenicity risks, restricted cargo capacity, and the potential for genotoxic insertional mutagenesis. In contrast, nanotechnology‐driven platforms provide advantages, including modularity, tunable surface functionality, and the ability to co‐deliver multiple therapeutic components within a single system [178]. Furthermore, nanomaterial‐enabled strategies facilitate non‐invasive, deep‐tissue optogenetic activation through mechanisms such as upconversion or photothermal effects, thereby circumventing the need for invasive optical fiber implants [60]. Nonetheless, nanoplatforms generally exhibit lower transfection efficiency than viral vectors, and critical issues regarding their long‐term biosafety, such as immunogenicity, off‐target organ accumulation, and degradation kinetics, remain inadequately characterized. Additionally, the low quantum yield of many nanomaterials necessitates a trade‐off between high nanoparticle doses and intense excitation power, which may lead to unintended phototoxicity [179]. Beyond biological and efficacy considerations, the clinical translation of these sophisticated nano‐optogenetic systems faces significant Chemistry, Manufacturing, and Controls (CMC) challenges. Scalable production of multi‐component nanotherapeutics requires stringent control over critical quality attributes, including particle size, surface charge, encapsulation efficiency, and batch‐to‐batch consistency [180]. The complexity of functionalization amplifies manufacturing hurdles and necessitates advanced characterization techniques to ensure product stability and reproducibility. Moreover, regulatory compliance for combination products demands rigorous validation of purity, sterility, and long‐term storage stability [181]. Addressing these CMC bottlenecks will require interdisciplinary collaboration among material scientists, process engineers, and regulatory experts to establish standardized protocols and accelerate the industrial translation of nano‐enabled optogenetic therapies.

**FIGURE 5:**
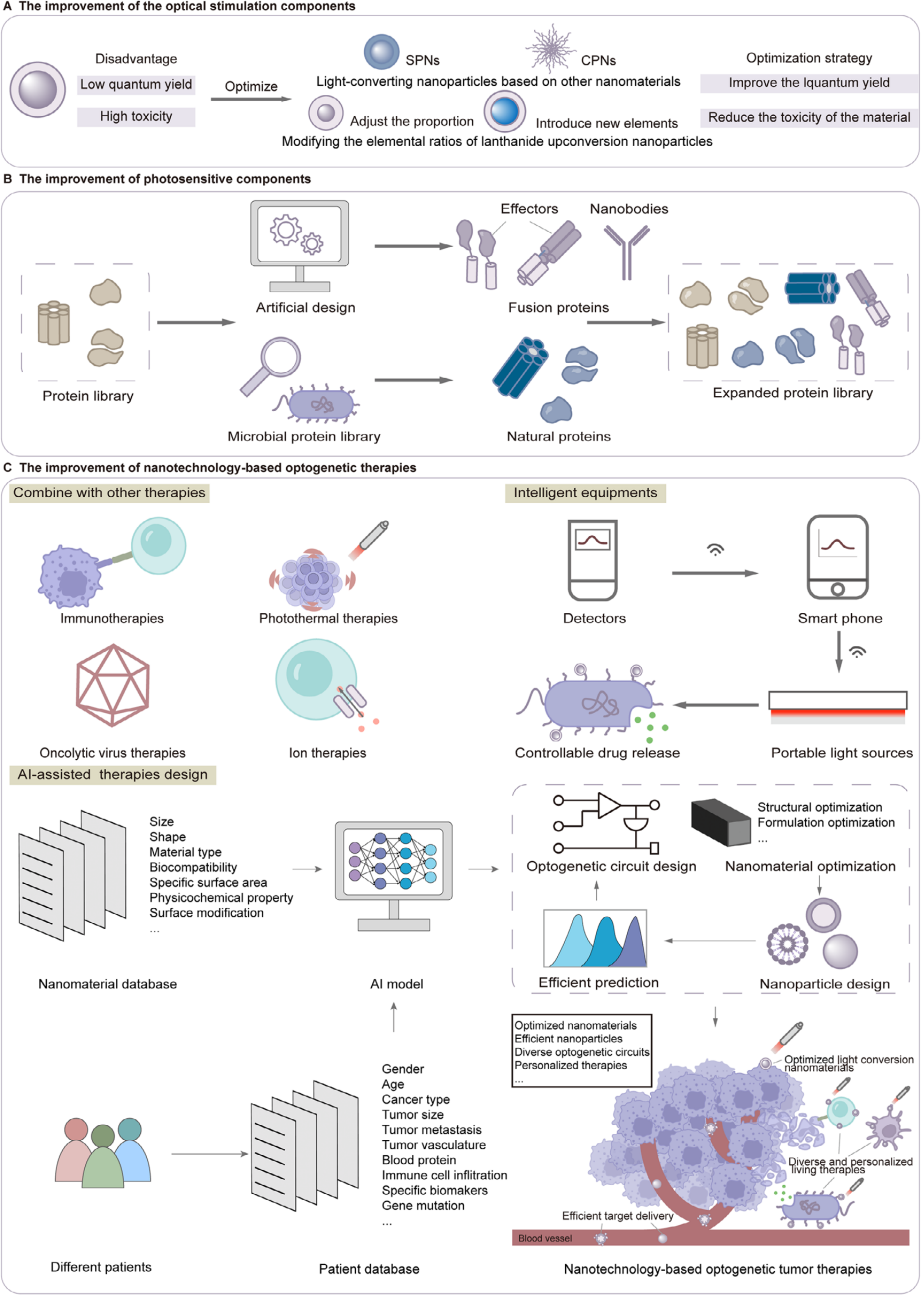
Potential strategies to overcome limitations of optogenetic therapy and promote the integration with nanotechnology. (A) The improvement of the optical stimulation components. The conventional lanthanide‐doped nanoparticles have low quantum yield and high toxicity. Changing the elemental composition or proportion of lanthanide‐doped nanoparticles can optimize quantum yield. Using other materials, such as light‐converting nanoparticles, can reduce toxicity. (B) The development of photosensitive components. Improvements in photosensitive components have focused on the design of fusion proteins or the discovery of more photosensitive proteins to expand the protein library. (C) The improvement of nanotechnology‐based optogenetic therapies. Combining with other therapies can enhance the therapeutic effect. Besides, intelligent devices can be introduced to realize the real‐time regulation of optogenetic therapy. The introduction of artificial intelligence can design an efficient nanocarrier by analyzing a nanomaterial database. By combining with data from patient sources, efficient and personalized nanotechnology‐based optogenetic tumor therapies can be achieved. SPNs, semiconductor nanoparticles; CPNs, conjugated polymer nanoparticles; AI, artificial intelligence.

To overcome these risks, three potential strategies are emerging. Firstly, composition optimization through adjusting elemental ratios and core‐shell architectures reduces non‐radiative energy loss, enabling lower laser intensities while maintaining optogenetic activation [182] (Figure 5A). Secondly, the size and geometry control of nanoparticles balance efficient cellular uptake with biosafety. The rational design of nanoparticle size is critical for balancing circulation longevity, tumor accumulation, and potential toxicity. Nanoparticles within the size range of approximately 20–200 nm are often considered, as they benefit from efficient accumulation in tumor tissues via the enhanced permeability and retention effect [183]. However, this design must carefully consider the trade‐offs associated with smaller dimensions; particles around 20 nm or smaller face challenges, including immune clearance and renal filtration, which can limit their tumor delivery efficiency [184]. Furthermore, such small nanoparticles may exhibit increased risks of nanotoxicity due to their characteristics, such as large specific surface area, non‐specific tissue penetration, and interactions with subcellular structures [185, 186]. Conversely, larger nanoparticles encounter restricted extravasation through tumor vasculature and are more susceptible to recognition and phagocytosis by the MPS, leading to rapid hepatic and splenic sequestration [183]. In addition, larger nanoparticles tend to accumulate in the MPS and may trigger pro‐inflammatory immune responses due to prolonged retention [184]. Consequently, the selection of nanoparticle size for optogenetic applications requires a multifaceted consideration. Anisotropic designs improve light harvesting efficacy while reducing excitation demands [187]. These strategies to manipulate the morphology of nanoparticles are expected to obtain low‐toxic and efficient optogenetic systems. Thirdly, emerging feedback‐regulated platforms that integrate real‐time monitoring with adaptive optogenetic modulation represent a novel method in reducing off‐target toxicity of optogenetics. With the help of fluorescence imaging and sensors, real‐time monitoring of optogenetic operations can be achieved [188]. Laser parameters can be adjusted according to the actual response to ensure the therapeutic effect while avoiding phototoxicity.

The development of wavelength‐specific photosensitive components faces two challenges: achieving wavelength‐selective activation while suppressing off‐target cellular responses. A strategy integrating natural protein discovery and computational de novo design shows potential to address these problems (Figure 5B). Nanopore sequencing enables rapid genomic analysis of deep‐sea organisms and microorganisms in extreme environments. Next, machine learning‐guided screening and in‐depth mining accelerate the discovery of long‐wavelength‐sensitive candidates [189]. Furthermore, AI‐augmented protein engineering enables the development of multi‐domain fused proteins. By using AI tools such as AlphaFold, researchers can accurately predict the structure and light‐responsiveness of fusion proteins, thereby improving the design efficiency of photosensitive proteins [190].

Finally, therapeutic protocol design must consider the patient‐specific heterogeneity (e.g., genomic instability and immune status) to maximize optogenetic outcomes. Combination therapies show great potential for improving the therapeutic efficacy of optogenetic therapies (Figure 5C). For instance, a near‐infrared‐activated nanosystem has been developed to enable light‐controlled CRISPR‐Cas9 gene editing while enhancing PDT in deep tumors. This dual activation achieved precise spatiotemporal control and synergistic anti‐tumor effects [191]. Combining optogenetic strategies with CAR T therapy addresses CRS via temporal activation control. Huang et al. split the CAR signaling domains into two parts, each fused with light‐responsive dimerization modules. Meanwhile, UCNPs were introduced as nano‐transducers to convert NIR light into blue light, driving the reassembly of split CAR to activate tumor‐killing functions [11]. In oncolytic virus therapies, engineered adenoviruses encoding photosensitive proteins achieve higher replication boost under blue light, enhancing tumor lysis while sparing normal tissues [10]. Combining optogenetics with ion therapy using MOFs and UCNPs also enables tumor‐specific ion influx with calcium‐induced apoptosis [20]. Furthermore, AI models, leveraging patient‐specific multi‐omics data and tumor spatial transcriptomics, not only predict optimal optogenetic actuator selection, wavelength regimens, and dosimetry calibration but also revolutionize optogenetic circuit design. By simulating complex biological interactions, AI algorithms can optimize light‐responsive genetic circuits for enhanced therapeutic. Moreover, AI‐driven design of delivery vectors improves the targeted delivery of optogenetic components, ensuring efficient gene transfer to tumor cells while minimizing off‐target effects. Smart intelligent devices represent another crucial direction for the improvement of optogenetic therapies. Implantable nano‐sensors can monitor the changes in various physiological indicators in real time and with high precision, which helps to adjust treatment parameters. For example, researchers have reported a portable smart blue‐light control device to regulate the expression of IFN‐*γ* and C‐X‐C motif chemokine 10 [192]. Besides, Ye et al. reported a far‐red light‐responsive gene regulation platform with wireless control. Their smartphone‐controlled system enables remote insulin secretion management in implanted photosensitive cells, showing efficacy in glycemic control [193]. Recent advances have further established bidirectional bio‐optical‐electronic signal processing chains using optogenetically engineered bacteria, ingestible optoelectronic capsules, and wireless smartphones, enabling real‐time diagnostic feedback and user‐controlled therapeutic interventions. For instance, Zhang et al. developed an integrated system where engineered E coli sensed inflammatory biomarkers such as nitrate and produced bioluminescent signals, which were detected by an ingestible capsule and wirelessly transmitted to a smartphone. Upon user command, the capsule emitted green light to activate the bacterial secretion of anti‐inflammatory nanobodies, effectively mitigating colitis in a porcine model. This closed‐loop platform highlights the potential of smart devices for precise, responsive, and minimally invasive optogenetic regulation in deep tissues [194].

Concurrently with addressing these technical challenges, the translation of laboratory advances into viable clinical therapeutics necessitates a thorough evaluation from the perspective of practical application. This process involves identifying the tumor types most likely to benefit from existing technological capabilities and selecting nanomedicine delivery systems that demonstrate high potential for successful clinical translation.

From a clinical perspective, certain tumor types are more amenable to optogenetics‐based therapies. Superficial or accessible malignancies, such as melanoma, head and neck cancer, and breast cancer, are particularly suitable due to the relative ease of light delivery [6]. Among these, melanoma has been extensively studied in preclinical optogenetics models, owing to its light‐responsive nature and superficial location, which facilitate non‐invasive optical activation [33]. Conversely, deep‐seated tumors (e.g., pancreatic or glioblastoma) present greater challenges for light penetration. However, the integration of NIR‐to‐visible upconversion nanomaterials or X‐ray‐stimulated scintillators may extend optogenetic applicability to these malignancies [8, 106]. Clinically, tumors with well‐defined margins and minimal metastatic burden may see the earliest translational success, as localized optogenetic control can be more effectively achieved.

Regarding NDDSs with high clinical translation potential, LNPs stand out due to their established use in messenger RNA vaccines, demonstrating scalability, biocompatibility, and regulatory acceptance [195]. LNPs offer efficient nucleic acid delivery, tunable surface functionalization, and potential for co‐delivery of optogenetic constructs. In comparison, polymer‐based nanoparticles also show promise due to their controlled release profiles and functional versatility, though their long‐term biodegradability and toxicity require further validation [196]. EVs represent another emerging candidate, with innate biocompatibility and low immunogenicity, yet manufacturing scalability and standardization remain hurdles. MNPs excel in deep‐tissue light conversion but face concerns regarding ion leakage and long‐term retention [197]. Thus, LNPs may offer the most viable path toward clinical adoption.

In summary, the integration of nanotechnology and optogenetics is emerging as a promising approach for precision cancer therapy. In the future, various technological advancements will continue to drive the clinical translation of optogenetics‐based cancer therapy. Novel NDDSs provide efficient and targeted delivery for optogenetic components. Diverse light‐conversion nanomaterials overcome the limitation of light penetration and enable deep‐tissue activation. Additionally, optogenetics‐based adoptive cell therapies offer programmable and spatiotemporally controlled antitumor functions. Moreover, AI will optimize optogenetics‐nanotechnology strategies by learning from large‐scale interdisciplinary datasets, providing insights for the rational design of light‐responsive systems. The convergence of these technologies will enrich the optogenetics toolbox, leading to precise and personalized cancer treatments.

## Author Contributions

H.S. and W.Y. wrote the manuscript and created the figures and tables. S.H., W.Y., X.Q., R.L., Q.Z., G.W., and D.Y. revised the initial manuscript and figures. W.Y. provided the conceptual idea and revised the manuscript. Y.L. provided financial supports and overall guidance. All authors have read and approved the final manuscript.

## Conflicts of Interest

The authors declare no conflicts of interest. Yaping Li is a member of the *Exploration* editorial board, and he was not involved in the handling or peer review process of this manuscript.

## Data Availability

Reprints and permissions information is available.
